# The Association of Internet Addiction with Burnout, Depression, Insomnia, and Quality of Life among Hungarian High School Teachers

**DOI:** 10.3390/ijerph19010438

**Published:** 2021-12-31

**Authors:** Marietta Pohl, Gergely Feher, Krisztián Kapus, Andrea Feher, Gabor Daniel Nagy, Julianna Kiss, Éva Fejes, Lilla Horvath, Antal Tibold

**Affiliations:** 1 Centre for Occupational Medicine, Medical School, University of Pécs, 7624 Pécs, Hungary; pohlmarietta@bmvk.hu (M.P.); kapusk@gmail.com (K.K.); julianna.kiss@shl.hu (J.K.); lilla.horvath@etk.pte.hu (L.H.); tibold.antal@pte.hu (A.T.); 2 Department of Primary Health Care, University of Pécs, 7623 Pécs, Hungary; 3 Szent Rafael Hospital, 8900 Zalaegerszeg, Hungary; fehergergelydr@gmail.com; 4 Department Of Social Studies, University of Szeged, 6722 Szeged, Hungary; nagy.gabor.daniel@szte.hu; 5 Hospital of Komló, 7300 Komló, Hungary; efchengirl@gmail.com

**Keywords:** internet addiction, burnout, depression, insomnia, quality of life, adult, teacher, cross-sectional study

## Abstract

The extensive availability of Internet has led to the recognition of problematic Internet use (so called Internet addiction, IA) mostly involving adolescents. There are limited data about the prevalence and consequences of IA in adults especially among high school teachers. Here, we present a cross-sectional prospective study focusing on the association of Internet addiction with burnout, depression, insomnia, and lower quality of life among high school teachers taking many co-variates into account. Overall, 623 males (34.3%) and 1194 females (65.7%) participated in our study. Internet addiction was detected in 5.2% (95/1817) based on the Problematic Internet Use Questionnaire. Internet addiction was associated with severe burnout (10.5 vs. 2.7%, *p* < 0.001), moderate (36.8 vs. 1.7%, *p* < 0.001), and severe (6.3 vs. 0.1%, *p* < 0.001) depression, insomnia (23.1 vs. 11.4%, *p* < 0.001), and severe sleep disturbance (severe insomnia, 27.4 vs. 3.8%, *p* < 0.001) and lower quality of life in all domains (*p* < 0.001). There was also a significant correlation of the severity of the above-mentioned parameters and the severity of IA (overall scores, *p* < 0.001 in all cases). In a multivariate analysis including demographic criteria, risk factors medical conditions and the above-mentioned parameters as co-variates internet addiction was significantly associated with depression (OR = 3.836, CI: 2.92–5.44, *p* = 0.03), and insomnia (OR: 3.932, CI: 3.6–5.69, *p* = 0.002). This is the first study from Hungary and is one of the first studies showing the association of IA with mental issues, burnout, and lower quality of life among adults. It underlines the clinical importance of problematic Internet use among adults.

## 1. Introduction

Internet addiction (IA or problematic Internet use) is a well-known phenomenon described almost three decades ago [1]. Similar to other types of addiction it is usually defined as prolonged problematic, compulsive use (of the Internet) with subsequent impairment in an individual’s function in various domains of life [2]. IA is not a single diagnosis and it should be regarded as an umbrella term covering all aspects of Internet misuse such as problematic gaming, online porn abuse, and social media overuse resulting in similar symptomatology; problematic users are not able to control their online activities, which lead to significant negative effects on their lives as mentioned above [3].

Despite intensive and cumulative research on the topic carried out in the last three decades, and due to a lack of consensus and scarcity of cross-sectional and absence of clinical studies, the identification of IA has been challenging [2].

Its prevalence is increasing over time and a recent review showed that approximately 7% of the whole population currently meet the criteria for Internet addiction [4].

IA has several risk factors such as gender (more prevalent in males), early exposure to Internet, low family income, and living in rural areas [5]. Particular personality traits such as sensation seeking, higher impulsivity, and lower self-control are strongly associated with the development of IA as well as neuroticism and being socially inactive, which can unbalance the ratio of offline and online activities [5,6]. Family functioning may also have a potential role as lack of family support or poor parent–adolescent relationships can be risk factors, while parental monitoring can be preventive [7].

Based on previously published meta-analyses of cross-sectional studies, IA can be associated with a wide range of social and mental issues such as substance abuse, depression, anxiety, poor sleep quality, and attention deficit hyperactivity disorder (ADHD); however; the association remains to be further investigated and clarified [8,9,10,11].

Poor sleep quality and duration have previously shown to be associated with IA in adolescents as a result of irregular sleep patterns with subsequent daytime sleepiness [12,13]. Internet addicts have a twofold risk to develop insomnia comparing to the general population [13].

IA also increases the risk of developing mental issues such as anxiety and depression [12]. There is an approximately 2.5-fold risk of having depression among problematic Internet users comparing to normal ones [14].

Despite of extensively growing literature, relatively few studies focused on the effect of IA on physical health [11]. Health-related quality of life seems to be lower among Internet addicts which can be related to the above-mentioned mental issues, but results are contradictory [11,15]. Recent studies have also showed the possible association of burnout and Internet addiction [16,17].

Burnout (similar to IA) is also an increasingly prevalent syndrome with different etiologies including both individual and on organizational perspectives [18]. Based on the generally accepted theory of Christina Maslach and her workgroup, burnout can be characterized by emotional exhaustion (deperition of both physical and emotional resources), depersonalization (having unreal feelings and thoughts), and reduced personal accomplishment (the feel of being incompetence and loss of productivity and achievement at work) [18,19]. Similar to Internet addiction, neither the definition nor the clinical classification of burnout is adequately clarified, it is still labelled as an occupational phenomenon [16].

Primarily, workplace factors are responsible for the development of burnout, but individual characteristics (personality traits) also play an important role. Pliancy and neuroticism (similar to IA) are also significant predictors of burnout [16,20].

The phenomenon has a considerable impact both on the individual and on the society as it may lead to undesirable consequences such as emotional depletion, loss of energy, dehumanization, detachment from work, feelings of inadequacy, reduced productivity and coping skills, and it may also be associated with mental and somatic complications such as depression, insomnia, cardiovascular disorders, and chronic pain syndromes [16,21]. Burnout can also lead to substance dependence such as tobacco, alcohol, or drugs or to the development of addictive behaviors such as problematic Internet use [22].

Problematic Internet use has been widely studied among adolescents but limited data are available on adult populations including the association of IA with burnout, depression, insomnia, and quality of life especially among teachers, who can be the first line of school-based prevention efforts [23,24]. We have previously examined the predisposing factors and prevalence of IA among teachers and here we present another cross-sectional, study focusing on the association of addiction to Internet, mental issues, and burnout on the same population [25].

The aim of our study was to analyze the role of risk factors (including detailed demographic data, substance abuse, most common medical conditions) and the above-mentioned mental issues and physical health parameters on problematic Internet use and to determine independent parameters associated with the phenomena.

## 2. Materials and Methods

### 2.1. Participants

This study was carried between January 2020 and August 2020 at 14 educational sites in Hungary (the names of the involved schools are listed in the Acknowledgement part). It was a cross-sectional prospective study in nature, applying paper-based questionnaires.

The study protocol and documentation were approved by the regional ethical committee (Ethics Committee of the University of Pecs, license number 8434-PTE 2020). Informed consent was read and signed by participants prior to delivery.

Inclusion criteria included being employed (public servant, subcontractor, etc.) as a high school teacher, aged between 18 and 65 years of age and being at work at the time of the study.

Exclusion criteria were refusing to participate in the study, being on permanent leave or being younger than 18 or being older than 65 years of age.

Included demographic data were age, gender, marital status, number of children, type of work, years spent with work, work schedule, legal relation, and secondary employment.

Risk factors and medical conditions included tobacco use, alcohol, and illicit drug use; the presence diabetes, hypertension, or ischemic heart disease; history of musculoskeletal pain and depression.

Daily time spent online, daily time interval and goals of Internet use were also collected.

### 2.2. Psychometric Measures

As there are no clear diagnostic criteria for Internet addiction, it is highly recommended to measure excessive Internet use with a continuous questionnaire [2]. We chose the problematic Internet use questionnaire (PIUQ) because its structure tightly adheres to the proposed diagnostic criteria for Internet addiction and was created based on the clinimetric and psychometric analysis of Young’s Internet addiction test independently validated by several groups and used in our previous published work [2,16,24,25]. The questionnaire contains 18 items, each scored on a 5-point Likert-type scale ranging from 1 (never) to 5 (always). A confirmatory factor analysis verified the three-factor model of the questionnaire, each subscale contains six items. Obsession subscale refers to obsessive thinking about the Internet (daydreaming, rumination, and fantasizing) and withdrawal symptoms caused by the lack of Internet use (anxiety and depression) (“How often do you feel tense, irritated, or stressed if you cannot use the Internet for as long as you want to?”). Neglect subscale contains items about neglecting everyday activities, social life, and essential needs (“How often do you spend time online when you’d rather sleep?”). Control disorder subscale reflects difficulties in controlling time spent on the Internet (“How often do you realize saying when you are online, “just a couple of more minutes and I will stop”?”). Since in this study we focused on global psychological consequences of Internet addiction, we used PIUQ total score in statistical analyses, which was computed by summing the scores on all the items of the scale. A total score exceeding 41 points suggests Internet addiction [25,26]. 

Burnout was measured with the Maslach Burnout Inventory (MBI), which is an introspective psychological inventory consisting of 22 items pertaining to three dimensions of burnout: emotional exhaustion, depersonalization, and personal accomplishment [19]. Responses are marked on a seven-point Likert scale (0 meaning “never” and 6 meaning “every day”) and then summed. The first nine items assess emotional exhaustion (EE), the second five item assess depersonalization (DP), the seven last items assess reduced personal accomplishment (PA). We defined EE as EE score ≥ 27, DP as DP score ≥ 10, and PA as PA score ≤ 33. The overall burnout was defined as EE score ≥ 27 and/or DP score ≥ 10 [27]. For EE and DP, subscale score of 0–9 was categorized as “no to low burnout” and subscale score of 10–18 was regarded as “moderate burnout.” It was the opposite for PA because higher PA scores indicate lesser burnout [19,27]. This copyright protected questionnaire can be downloaded in Hungarian translation from the website.

Depression was detected the short version of Beck Depression Inventory (BDI-SF), which examines the severity of depression using 9 questions and demonstrated good internal consistency in Hungarian samples [28,29]. The questionnaire asks about the following symptoms: social withdrawal, indecision, sleep disturbance, fatigue, excessive anxiety about physical symptoms, incapacity for work, pessimism, dissatisfaction, lack of joy, self-blame, each item scored from 1 to 4 points. After summarizing the results, we can distinguish between severe (≥26 points), moderate (19–25 points), mild depression (10–18 points), or the absence of mood disorder (0–9 points) [29].

Sleep disturbance was measured with Athens Insomnia Scale (AIS) [30]. The questionnaire contains eight items about nocturnal symptoms (difficulty of falling asleep, early awakening), and three items about daytime consequences. The higher the score, the worse the quality of sleep (maximum 24 points) Having >6 points means insomnia, while >10 points indicates clinically significant sleep disturbance (severe insomnia) [30].

Quality of life is measured by the EQ-5D (health-related quality of life), which is a self-administered questionnaire measuring 5 dimensions of everyday life (mobility, self-sufficiency, normal daily activities, pain/malaise, and anxiety/depression) with a 3-point Likert- scale [31].

### 2.3. Statistical Analysis

Data were evaluated as means ± SD (standard deviation) by Student’s *t*-test, the chi square test and the Pearson’s rank-order correlation.

Correlation model included the total score of PIU-Q (Internet addiction) as dependent variable, independent variables were the total scores of MBI, BDI-SF, AIS, and EQ-5D questionnaires.

Logistic regression analysis was used to determine the significance of the different parameters as independently associated with IA. The analysis included demographic factors (age, gender, marital status, number of children, type of work, years spent with work, work schedule, legal relation, and secondary employment), medical parameters (tobacco use, alcohol and illicit drug use; the presence diabetes, hypertension or ischemic heart disease; history of musculoskeletal pain and depression), time and purpose of Internet use (daily time spent online, daily time interval, and goals of Internet use) as well as burnout, depression, sleep disturbance and quality of life. The analysis was performed with appropriate adjustments for differences in risk factors and medication usage. For all odds ratios, an exact confidence interval (CI) of 95% was constructed in our study. Data analysis was performed using SPSS (version 22.0, IBM, New York, NY, USA).

## 3. Results

Overall, 1817 responses were received from the 2500 delivered questionnaire, which means a response rate of 72.7%. 623 (34.3%) males and 1194 females (65.7%) were included into our analysis.

Based on the results of PIU-Q the rate of Internet addiction was detected in 5.2% (95/1817) in the study population. The association of IA with baseline characteristics, demographic data and risk factors have previously been published [19].

In our study population, 26.0% (473/1817) suffered from mild, 70.9% (1288/1817) from moderate, and 3.1% (56/1817) from severe burnout based on the Maslach Burnout Inventory. Internet addiction was associated with severe burnout (10.5 vs. 2.7%, *p* < 0.001) (Table 1). There was a weak, but significant correlation between the severity of Internet addiction and burnout (overall scores) (r^2^ = 0.2, *p* < 0.001) (Table 2).

Regarding the subcategories, mean values were the following: emotional exhaustion 22.1 ± 9.1 points, depersonalization 20.9 ± 6.9 points, personal accomplishment 9.8 ± 4.6 points. Internet addiction was associated with more severe emotional exhaustion (25.6 ± 10.9 vs. 21.9 ± 8.9 points, *p* < 0.001) and depersonalization (12.7 ± 5.9 vs. 9.8 ± 4.5 points *p* < 0.001) but not with personal accomplishment (Table 1).

Depression could not be detected in 37.1% (673/1817) of the participants, while 58.9% (1070/1817) had mild, 3.5% (65/1817) had moderate and 0.6% (9/1817) had severe depression based on the results of BDI. Internet addiction was significantly associated with moderate (36.8 vs. 1.7%, *p* < 0.001) and severe (6.3 vs. 0.1%, *p* < 0.001) depression (Table 1). Furthermore, there was a significant correlation between the severity of IA and depression (overall scores) (r^2^ = 0.558, *p* < 0.001) (Table 2).

Insomnia could be found in 17.1% of the study population (311/1817), while 5.0% (92/1817) suffered from severe sleep disturbance. Internet addiction was associated with insomnia (23.1 vs. 11.4%, *p* < 0.001) and severe sleep disturbance (severe insomnia, 27.4 vs. 3.8%, *p* < 0.001) (Table 1). The severity of insomnia was also significantly correlated with PIU-Q overall scores (r^2^ = 0.325, *p* < 0.001) (Table 2).

Internet addiction was associated with lower quality of life taking all subcategories into account (*p* < 0.001 in all cases) (Table 1). There was a weak, but still significant correlation between these subscales and the severity of IA (*p* < 0.001 in all cases) (Table 2).

In a multivariate analysis including all factors (demographic data, internet habits, comorbidity, etc.), age < 35 years (OR: 6.098, CI: 5.09–7.08, *p* < 0.001), male gender (OR = 5.413, CI: 4.39–6.18, *p* = 0.002), surfing on the internet > 5 h daily (OR 2.568, CI: 2.03–3.39, *p* < 0.001), having no children (OR: 1.353, CI: 1.13–1.99, *p* = 0.0248), and having secondary employment (OR = 11.377, CI: 8.67–13.07, *p* = 0.001) were significantly associated with internet addiction. Depression (OR = 3.836, CI: 2.92–5.44, *p* = 0.03), and insomnia (OR: 3.932, CI: 3.6–5.69, *p* = 0.002) were also strongly correlated with IA (Table 3).

## 4. Discussion

Internet addiction has been extensively studied among adolescents, but limited data are available on adult populations especially with emphasis on the association of burnout and mental issues.

The overall prevalence of Internet addiction is about 7% in the whole population with younger (adolescent) predominance; the 5% rate of IA in our study is comparable to these findings and similar to the results of other publications including adult populations in Hungary [4,16,17].

Internet addiction can be associated with mental symptoms such as depression and insomnia. Depression (especially severe depression) is a disabling state for the individual and a great burden on the society; it is projected to be the leading cause of disability by 2030 and one of the leading causes of death due to increased suicide rates. Recent studies have showed the association of depression and IA among adolescents, but data are missing on adult populations [32]. Our study showed an increased rate of moderate and severe depression among problematic Internet users and the severity of depression was significantly correlated with the severity of problematic Internet use. Depression remained a significant parameter associated with Internet addiction based on multivariate analysis; however; causality is not entirely clarified. Based on recent findings summarized in a meta-analysis, IA had significantly higher, at least threefold, rates of suicidal ideation, planning, and attempts which underlines the of screening and prevention [33].

The association of IA and depression is not well-understood. Personality traits and prior depression may have a significant effect on the development of Internet addiction or they may enhance each other. Internet addiction may also lead to depression as a consequence of addictive behavior [2,16,34]. In our previous study, history of depression was not associated with IA which raises the possibility of Internet addiction and subsequent depression, but it merits further investigation [25].

Insomnia can be the consequence of longer Internet use in adolescent samples, but its association with IA has been poorly studied in adult populations [35,36]. Similar to adolescent samples, both insomnia and severe insomnia were associated with problematic Internet use in our study in both uni- and multivariate analysis. The underlying pathophysiology is also not well-understood, insomnia can lead predispose to nighttime Internet use with subsequent addiction or problematic Internet use or nighttime Internet use (nighttime Internet use is one of the strongest predictors of addiction) that can result in poor sleep quality [25,36]. In our study nor the goal neither the time of Internet use were associated with IA, which may suggest that problematic Internet use is the predecessor of sleeping problems as there was a significant correlation between the severity of problematic Internet use and sleeping problems, but it should be treated with caution due to the nature of our study (cross-sectional).

The association of burnout and IA is also rarely documented. Burnout may be associated with problematic Internet use among healthcare professionals, but results are conflicting [16,37]. In a nationwide Japanese study, burnout was significantly associated with at-risk IA, but Internet addiction was not found in the sample population based on the applied questionnaire [17]. We found higher prevalence of severe burnout among problematic Internet users and higher points on emotional exhaustion and depersonalization scales as well as a weak, but significant correlation between IA and burnout overall scores. Based on recent publications, emotional exhaustion can lead to anxiety and impaired communications skills with subsequent social isolation as well as depersonalization, which can result in turning to the Internet as a coping method leading to IA [16,17]. However; burnout was not a significant predictor of Internet addiction based on a multivariate analysis.

Internet addiction can be associated with reduced physical activity, obesity, chronic pain syndromes, mental issues, disturbed circadian rhythm as well as with emotional and social problems which can be associated with low quality of life [38,39]. In our study population, IA was found to be associated with depression, insomnia and burnout that can be responsible for reduced quality of life among Internet addicts in all aspects of the questionnaire domains. There were weak, but still significant correlation among problematic Internet use and the above-mentioned subscales. Interestingly, quality of life was not significantly associated with IA in a multivariate analysis.

## 5. Conclusions

This is among the first studies focusing on the association of Internet addiction with depression, insomnia, burnout, and quality of life among adults. One out of twenty teachers suffered from IA (which is a pretty high rate) and strong association was found among the examined parameters raising the possibility that problematic Internet use is more than just a phenomenon of mental instability of adolescents.

Finally, our article has some limitations. Although it was a prospective study including a large number of high-school teachers, it was not representative of Internet addiction neither among teachers nor in the adult population. It was a cross-sectional study; therefore, causalities could not be entirely clarified. Due to the nature of our study, we could not include clinical data in our analysis such as detailed information about the participants’ socio-economic status, medical history (type and duration of medical disorders), and physical examination or follow-up were not carried out. The above-mentioned limitations may have influenced our findings.

## Figures and Tables

**Table 1:** Comparison of burnout, depression, sleep disturbance, and quality of life among the subgroups.

	Not Addicted to Internet (n = 1722)	Internet Addiction (n = 95)
**Burnout**		
low	455 (26.4%)	18 (18.9)
moderate	1221 (70.9%)	67 (70.5%)
severe	46 (2.7%)	10 (10.5%) **
emotional exhaustion	21.9 ± 8.9	25.6 ± 10.9 **
depersonalisation	9.8 ± 4.5	12.7 ± 5.9 **
personal accomplishment	20.9 ± 6.9	21.2 ± 8.9
**Depression**		
no depression	665 (38%)	8 (8.4%)
mild	1024 (59.5%)	46 (48.4%)
moderate	30 (1.7%)	35 (36.8%) **
severe	3 (0.1%)	6 (6.3%) **
**Sleep disturbance**		
no	1459 (84.7%)	48 (50.5%)
insomnia	197 (11.4%)	22 (23.1%) **
severe insomnia	66 (3.8%)	26 (27.4%) **
**Quality of life (points)**		
mobility	1.23	1.81 **
self-sufficiency	1.45	2.33 **
daily activities	1.21	1.95 **
pain/dyscomfort	1.29	1.68 **
anxiety/depression	1.18	1.50 **

** *p* < 0.001.

**Table 2:** Correlation between internet addiction with burnout, depression, insomnia, and quality of life subcategories (PIUQ: Problematic Internet Use Questionnaire, MBI: Maslach Burnout Inventory, AIS Athens Insomnia Scale).

		MBI	BECK	AIS	Mobility	Self-Sufficiency	Daily Activities	Pain
**PIU-Q**	Pearson Correlation	0.200	0.558	0.325	0.143	0.266	0.263	0.181
	*p* value	<0.001	<0.001	<0.001	<0.001	<0.001	<0.001	<0.001

**Table 3:** Risk factors associated with internet addiction in a multivariate analysis.

Risk Factor	OR	CI
age < 35 years	6.098	5.09–7.08 **
male gender	5.413	4.39–6.18 *
>5 h daily internet use	2.568	2.03–3.39 **
having no children	1.353	1.13–1.99 *
having secondary employment	11.377	8.67–13.07 *
Current depression	3.836	2.92–5.44 *
Insomnia	3.932	3.6–5.69 **

* *p* < 0.05, ** *p* < 0.001.

## Data Availability

The dataset supporting the conclusions of this article is available on request to the corresponding author.
